# Riding Feats

**Published:** 1890-02

**Authors:** 


RIDING FEATS.—A number of officers of Magdeburg Hussars, No. 10, on June 28th, marched from Stendal to Hanover, 162 kilometres (101 miles), in eighteen hours (including two-and-a-half hours halt). They started at 2 A.M., and arrived at 8 P. M. They returned on July 1st, and arrived with men and horses in good condition.

First Lieutenant Seiffert (1st Uhlans) in five days rode from Spandau to Militsch, in Silesia, on a 7 year old English horse, not especially trained, 65.70 kilometres *per diem*. The weather was very hot. (*Militar Wochenblatt.*)

